# Tofacitinib as an adjuvant treatment for pediatric Still's disease

**DOI:** 10.3389/fped.2025.1650675

**Published:** 2025-08-20

**Authors:** Ling Hou, Peng Zhou, Chengguang Zhao, Xiuli Wang, Yue Du

**Affiliations:** Department of Pediatrics, Shengjing Hospital of China Medical University, Shenyang, China

**Keywords:** JAK-STAT, janus kinase inhibitor, pediatric Still’s disease, systemic juvenile idiopathic arthritis, tofacitinib

## Abstract

**Objective:**

To describe the efficacy and safety of tofacitinib for pediatric Still's disease, also referred to as systemic-onset juvenile idiopathic arthritis (sJIA). Traditional non-biological drugs and drugs targeting the interleukin-1 and interleukin-6 pathways benefit some patients, but others show inadequate responses.

**Methods:**

We retrospectively analyzed ten patients with pediatric Still's disease who were treated with tofacitinib and had at least one follow-up visit. Data on patient history, laboratory findings, and treatments were collected at disease onset, at the initiation of tofacitinib, and during follow-up.

**Results:**

Tofacitinib led to complete remission in six patients, partial remission in three patients, and loss of efficacy in one patient. Among the nine patients with remission, two discontinued corticosteroids entirely and seven used lower dosages of corticosteroids; these patients also used fewer concurrent medications (1 or 2) after tofacitinib initiation. The one patient who experienced loss of efficacy continued to require a higher dosage of corticosteroids and received five different additional medications. The other nine patients received tocilizumab and had a decreased frequency of these injections after tofacitinib initiation. Tofacitinib was well-tolerated, with only one reported instance of an upper respiratory tract infection.

**Conclusions:**

Tofacitinib appears to be an effective adjunct therapy for management of pediatric Still's disease, particularly for patients with unstable clinical conditions and adverse reactions to corticosteroids.

## Introduction

Pediatric Still's disease is a rare but severe subtype of juvenile idiopathic arthritis (JIA) that primarily affects children under 6-years-old. In addition to arthritis, these patients also develop fever and rashes, and may also experience generalized lymphadenopathy, hepatosplenomegaly, and serositis (1). Pediatric Still's disease can trigger a systemic inflammatory response (SIR) which can lead to life-threatening complications, such as macrophage activation syndrome (MAS) (1). Despite the availability of various treatments, including non-biological disease modifying anti-rheumatic drugs (DMARDs) and biological DMARDs that target the interleukin-1 (IL-1) and interleukin-6 (IL-6) pathways, about 15% of these patients respond poorly to these therapies or experience relapses (2).

Tofacitinib is an oral immunomodulator that has promising efficacy and safety when used for treating adult rheumatoid arthritis (RA) and other autoimmune diseases (3, 4). Tofacitinib is also effective in treating polyarticular JIA, thus sparing children the discomfort of intra-articular injections (5). At the molecular level, tofacitinib inhibits Janus kinases (JAKs), non-receptor intracellular tyrosine kinases that are responsible for adaptive and innate immune responses in immune-mediated inflammatory diseases, thus reducing the production of inflammatory cytokines (6, 7). Previous research led the U.S. FDA to approve tofacitinib for treatment of RA (April 2012) and polyarticular JIA (September 2020). Tofacitinib is not yet approved for pediatric Still's disease, although ongoing Phase III clinical trials are examining the use of tofacitinib (NCT03000439) and baricitinib (another JAK inhibitor, NCT04088396) for treatment of this disease (8).

In this study, we described the potential efficacy and safety of tofacitinib for patients with pediatric Still's disease by conducting a retrospective study at Shengjing Hospital (SJH) of China Medical University from 2017 to 2023. Our analysis examined the detailed clinical characteristics and laboratory results before and after tofacitinib treatment and the records of adverse effects after treatment to determine the therapeutic benefits and safety of this medication. We focused on the benefits of tofacitinib for patients with pediatric Still's disease who had inadequate responses to non-biological and biological DMARDs. Furthermore, we examined the potential of tofacitinib to decrease the use of other treatments, such as oral corticosteroids, whose long-term use can have adverse effects, and the frequency of tocilizumab injections, which are uncomfortable for most patients.

## Materials and methods

### Patient selection

Consecutive patients who were diagnosed with pediatric Still's disease and received tofacitinib treatment at the Pediatric Rheumatology Department of SJH of China Medical University between January 2017 and December 2023 and had at least one follow-up visit were included. These patients also received concurrent treatment with corticosteroids and other anti-rheumatic drugs. The diagnosis of pediatric Still's disease was according to the 2001 International League Against Rheumatism criteria for JIA (9) or the 2019 classification criteria from the Pediatric Rheumatology International Trials Organization (10).

### Data collection

Data were collected retrospectively and included basic patient demographics (gender, age, body weight), disease duration, clinical symptoms (fever, rash, arthritis or arthralgia, enlarged lymph nodes, hepatosplenomegaly, serositis, and the presence of MAS), and laboratory results [white blood cells, neutrophils, platelets, C-reactive protein [CRP], erythrocyte sedimentation rate [ESR], ferritin, and liver enzymes]. These data were collected at the onset of the disease, at the initiation of tofacitinib treatment, and 1, 3, 6, 9, 12, 18, and/or 24 months after initiation of tofacitinib. The history of prior treatments, including corticosteroids and anti-rheumatic drugs, was also recorded.

### Assessment of tofacitinib efficacy and adverse reactions

The responses to tofacitinib treatment were categorized as complete remission [full resolution of all clinical symptoms [fever, rash, joint pain, arthritis, enlarged lymph nodes, hepatosplenomegaly, serositis] and laboratory markers [complete blood count, CRP, ESR, ferritin]]; partial remission (partial alleviation of clinical symptoms, but with some persisting symptoms or abnormal lab results); or treatment failure (no improvement in clinical symptoms or lab results) after 6 months of treatment onset. The loss of efficacy was defined as worsening of clinical symptoms and laboratory indicators following initial improvement. All adverse events were meticulously recorded at each follow-up visit.

### Ethics

Patients and their parents were fully informed that tofacitinib was approved by the U.S. FDA for use in adults with RA and for children over 2-years-old who have polyarticular JIA, although it is not yet approved in China for pediatric polyarticular JIA or pediatric Still's disease. Consent forms were obtained from all patients and their parents prior to treatment, and were recorded in the medical records. This study was reviewed and approved by Ethics Committee of Shengjing Hospital of China Medical University (2025PS641K).

## Results

### Characteristics of patients at onset of pediatric still's disease and initiation of tofacitinib

We examined seven male and three female patients. The median age at diagnosis of pediatric Still's disease was 6.00 years (range: 1.33–13.00 years), the median age at initiation of tofacitinib treatment was 9.02 years (range: 1.58–14.92 years), and the disease duration prior to initiation of tofacitinib was 1 month to 4.33 years (0.08 –4.33 years) (Table 1). All patients had symptoms of systemic disease at presentation, including fever, rash, arthralgia, or enlarged lymph nodes. Four patients had MAS before initiation of tofacitinib.

**Table 1:** Characteristics of patients at disease onset and at initiation of tofacitinib treatment*.

	Disease onset	Tofacitinib initiation
Demographic characteristics		
Age (years)	6.00 (1.33, 13.00)	9.02 (1.58, 14.92)
Sex, male: female	7:3	7:3
Clinical characteristics		
Fever (>39 °C)	9/10	6/10
Rash	9/10	4/10
Arthritis/arthralgia	7/10	2/10
Lymphadenopathy	6/10	1/10
Serositis (pericarditis, pleuritis)	2/10	0/10
Complications^a^	4/10	0/10
Laboratory characteristics		
Leukocytes (×10^9^/L)	16.08 (9.01, 35.93)	14.64 (10.05, 23.27)
Neutrophils (×10^9^/L)	12.30 (6.30, 30.20)	12.70 (4.60, 19.90)
Platelets (×10^9^/L)	385.50 (196.00, 563.00)	351.00 (172.00, 563.00)
CRP, mg/L	113.50 (76.50, 330.00)	19.55 (1.00, 171.00)
Ferritin, ng/ml	2,154.00 (143.60, 6,423.00)	222.15 (18.00, 15,000.00)
ESR, mm/h	61.50 (33.00, 100.00)	30.50 (2.00, 100.00)
Elevated liver transaminase	1/10	0/10
Previous treatment		
MTX	5/10	4/10
TNF inhibitor	0/10	0/10
IL-6 inhibitor^b^	8/10	8/10
Other treatments		
Cyclosporin A	5/10	3/10
Etoposide	4/10	1/10
Hydroxychloroquine	2/10	1/10
Colchicine	1/10	0/10

* Values indicate n/N or median (range).

^a^ Macrophage activation syndrome.

^b^ Tocilizumab.

MTX, methotrexate; CRP, C-reactive protein; ESR, erythrocyte sedimentation rate.

At disease onset, all patients had evidence of significant disease activity (Tables 1, 2). The median CRP level was 113.50 mg/L (range: 76.50–330.00; normal range: 0–8), the median ferritin level was 2,154.00 ng/ml (range: 143.60–6,423.00; normal range: 11–336.2), and the median ESR was 61.50 mm/h (range: 33.00–100.00; normal range: 0–20). At the initial presentation, one patient (No. 1) had pericardial effusion, one patient (No. 9) had pleural effusion, and one patient (No. 3) had elevated liver enzymes. At the initiation of tofacitinib treatment, the elevated liver enzymes in patient No. 3 had returned to normal; five patients (Nos. 1, 2, 3, 4, and 6) had marked inflammation with elevated levels of CRP (29.2–177 mg/L); six patients (Nos. 1, 2, 4, 7, 8, and 10) had recurrent fever; four patients (Nos. 2, 5, 6, and 10) had recurrent rash; two patients (Nos. 6 and 7) had arthritis; and one patient (No. 9) required a lower corticosteroid dose due to steroid-induced growth retardation. Eight patients (Nos. 1, 3, 4, 5, 7, 8, 9, and 10) were taking three or more other anti-rheumatic drugs.

**Table 2:** Characteristics of patients before initiation of tofacitinib and at the last follow-up.

No.	Sex	Pediatric Still's disease duration at tofacitinib initiation (years)	Age at tofacitinib initiation (years)	Treatments before tofacitinib	Tofacitinib dosage	Response at last follow-up	Concomitant DMARDs at last follow-up	Tofacitinib use at last follow-up (months)	CRP at tofacitinib initiation (mg/L)	CRP at last follow-up (mg/L)	Prednisone at tofacitinib initiation (mg/kg/day)	Prednisone at end of follow-up (mg/kg/day)	Remaining symptoms
1	M	0.25	12.25	Prednisone Cyclosporin A Etoposide	7.5 mg qd	Complete remission	Cyclosporin A	Ongoing (24)	77.3	1.9	0.85	0	None
2	F	1.04	11.04	Prednisone Tocilizumab	5 mg bid	Complete remission	Tocilizumab	Ongoing (24)	29.2	5	0.21	0.04	None
3	M	3.71	7.71	Prednisone Tocilizumab Etoposide MTX Colchicine HCQ	2.5 mg qd	Loss of efficacy	MTX Tocilizumab HCQ Colchicine	Ongoing (21)	171	118	0.40	0.24	Arthritis Elevated CRP
4	M	1.92	14.92	Prednisone Cyclosporin A Tocilizumab HCQ	5 mg qd	Complete remission	Tocilizumab	Stopped (24)	155	5	0.06	0	None
5	M	4.33	10.33	Prednisone Cyclosporin A Tocilizumab MTX Etoposide	5 mg qd	Partial remission	Tocilizumab	Ongoing (24)	2.47	33.27	0.07	0.07	Elevated CRP
6	M	0.08	6.00	Prednisone MTX	2.5 mg bid	Partial remission	MTX Tocilizumab	Ongoing (12)	76.5	5	1.63	0.09	Morning stiffness
7	F	0.25	1.58	Prednisone Cyclosporin A Tocilizumab	2.5 mg bid	Partial remission	Colchicine Thalidomide	Stopped (9)	9.9	1	0.087 (Dexamethasone)	0.014 (Dexamethasone)	Arthralgia
8	M	2.08	6.08	Prednisone Tocilizumab MTX Celecoxib	2.5 mg bid	Complete remission	Tocilizumab Celecoxib	Ongoing (12)	2.9	5	0.04	0.09	None
9	M	0.58	3.16	Prednisone Tocilizumab MTX	2.5 mg bid	Complete remission	Tocilizumab	Ongoing(12)	1	5	0.25	0.06	None
10	F	0.08	13.08	Prednisone Cyclosporin A Tocilizumab Etoposide	5 mg bid	Complete remission	Tocilizumab	Ongoing (9)	6.6	5.14	0.93	0.10	None

CRP, C reactive protein; DMARD, disease modifying anti-rheumatic drug; HCQ, hydroxychloroquine; MTX, methotrexate.

### Efficacy and adverse reactions to tofacitinib

**Figure 1:**
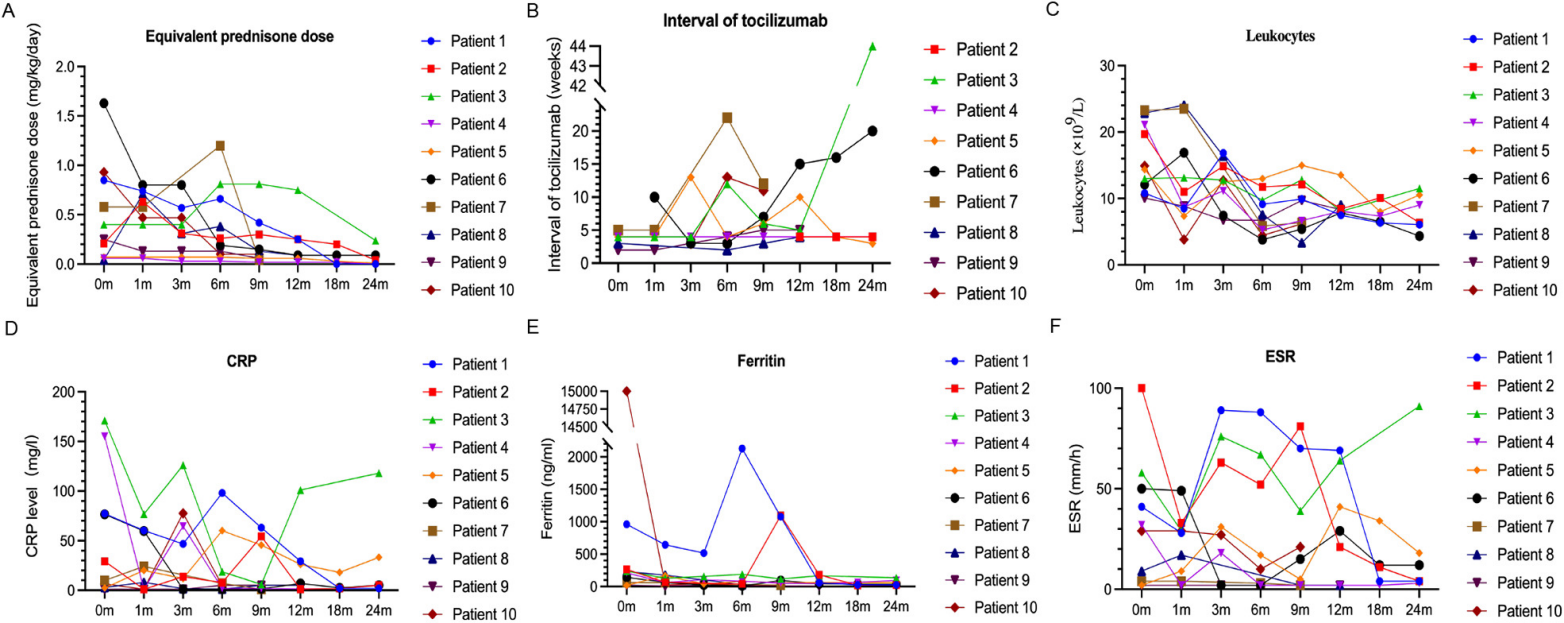
Changes in the dosage of prednisone **(A)** and frequency of tocilizumab injections **(B)**, and in the levels of leukocytes **(C)**, CRP **(D)**, ferritin **(E)**, and ESR **(F)** during follow-up.

Six of the patients (Nos. 1, 2, 4, 8, 9, and 10) achieved complete remission (Table 2 and Figure 1), as indicated by the absence of clinical manifestations and normalization of laboratory results. Three of these patients (Nos. 1, 2, and 4) received follow-up for 24 months, two patients (Nos. 8 and 9) received follow-up for 12 months, and one patient (No. 10) received follow-up for 9 months. At the last follow-up, patient No. 4 had discontinued tofacitinib and prednisone, and patient No. 1 had stopped prednisone. Thus, as of the last follow-up, the ten patients used 1 or 2 types of medications, primarily tofacitinib combined with tocilizumab, with supplementary low-dosage prednisone (<0.1 mg/kg/day).

As of the last follow-up, three patients (Nos. 5, 6, and 7) achieved partial remission (Table 2 and Figure 1); patient No. 5 had elevated levels of CRP and ESR, but no clinical symptoms; and patients No. 6 and No. 7 had joint symptoms but normal lab results. All three of these patients (No. 5, 6, and 7) had significantly lower prednisone dosages (<0.1 mg/kg/day). At the family's request, patient No. 7 discontinued tofacitinib at 9 months due to instability in her condition.

Patient No. 3 experienced an apparent loss of tofacitinib efficacy (Table 2 and Figure 1). His levels of CRP and ESR were low at the 9-month follow-up, but these levels subsequently increased, as did the symptoms of arthritis. This patient required a higher dosage of prednisone (0.24 mg/kg/day) and treatment with other medications (methotrexate, tocilizumab, hydroxychloroquine, colchicine), but continued using tofacitinib at the family's request.

### Corticosteroid Use after initiation of tofacitinib

Following initiation of tofacitinib, all patients except Nos. 3 and No. 7 had lower daily prednisone dosages (Table 2 and Figure 1). Two patients (Nos. 1 and 4) completely discontinued prednisone, and six other patients (Nos. 2, 5, 6, 8, 9, and 10) used a prednisone dosage below 0.1 mg/kg/day. Because steroid use can slow the growth rate of children, the prednisone dosage of patient No. 9 was decreased from 0.25 to 0.06 mg/kg/day after starting tofacitinib. Despite initial instability following decreases in the prednisone dosage in two patients (Nos. 3 and 7), both of these patients were using lower dosages as of the last follow-up.

### Frequency of tocilizumab injections after initiation of tofacitinib

In addition to tofacitinib, nine patients (all except No. 1) received tocilizumab. The duration of tocilizumab therapy was over one year in five patients (Nos. 2, 3, 4, 5, and 8), three months in patient No. 7, and four months in patient No. 9. Patients No. 6 and No. 10 developed MAS early during the course of disease, and because this condition was difficult to control, we administered tofacitinib one month after initiating tocilizumab. Following the initiation of tofacitinib, the administration intervals for tocilizumab were extended beyond the standard two-week recommendation (Figure 1). In the complete remission group (*n* = 6), patients Nos. 2 and 4 had intervals of four weeks, patient No. 8 had intervals from two to four weeks, patient No. 9 had intervals from two to five weeks, and patient No. 10 had intervals of three to thirteen weeks. In the partial remission group (*n* = 3), patient No. 5 had intervals of three to thirteen weeks, patient No. 6 had intervals of three to twenty weeks, and patient No. 7 had intervals of five to twenty-two weeks. Patient No. 3 (who experienced a loss of tofacitinib efficacy) had intervals of four to twelve weeks during the first 12 months prior to tocilizumab discontinuation.

### Tolerability and adverse reactions

The patients generally had good toleration of the tofacitinib treatments. However, patient No. 3 experienced a minor upper respiratory tract infection (nasal congestion and mild cough). There were no reports of severe infections, elevated liver enzymes, elevated serum creatinine, cardiovascular events, or cancer.

## Discussion

Pediatric Still's disease is a complex form of JIA in which patients typically present with fever, rash, arthritis, lymphadenopathy, hepatosplenomegaly, and/or serositis (1). Pediatric Still's disease accounts for approximately 10% of all JIA cases in North America (11), but as many as 50% of all cases in some Asian countries (12). Because pediatric Still's disease is characterized by myriad complications and chronic immune suppression, it is associated with the highest mortality rate among all JIA subtypes. The standard treatment regimen for pediatric Still's disease typically includes non-steroidal anti-inflammatory drugs (NSAIDs), corticosteroids, and/or DMARDs. Treatment with biological DMARDs that target IL-1 and IL-6 has led to improved prognosis for these patients, although a small subset of patients exhibit poor responses or relapses when given these newer treatments (2).

Extensive research has documented the efficacy of oral JAK inhibitors, such as tofacitinib and baricitinib, for adult RA, psoriatic arthritis, and other conditions (3, 4, 13–16). These drugs interfere with the signaling pathways of various cytokines, such as IFN-*γ*, IL-2, IL-6, IL-10, IL-12, IL-23, and GM-CSF (17). Tofacitinib is primarily a JAK1 and JAK3 inhibitor that is approved by the U.S. FDA for treatment of adults with RA that is resistant to methotrexate (13). Similar to biological DMARDs, tofacitinib is associated with an increased risk of infection and can also lead to other adverse effects, such as increased LDL cholesterol, neutropenia, and elevated liver enzymes (18, 19). Pediatric studies (5) demonstrated that tofacitinib was effective and well-tolerated in children with polyarticular JIA, with no adverse effects, and that the grape-flavored solution was well-received by patients. In 2020, the U.S. FDA approved tofacitinib for treatment of active polyarticular JIA in patients aged 2 years and older (5). Ongoing phase III clinical trials are currently evaluating the application of tofacitinib for pediatric Still's disease.

Our retrospective cohort study examined ten patients with pediatric Still's disease who had insufficient responses to non-biological and biological DMARDs and received subsequent treatment with tofacitinib. The results indicated that six patients achieved complete remission of clinical symptoms, normalization of laboratory indicators, and significant reductions in the number of concurrent medications (1 or 2 in addition to tofacitinib); two of these six patients completely discontinued corticosteroids, and all six used substantially lower dosages; five of these six patients received combination therapy with tocilizumab, and received less frequent injections of tocilizumab after initiation of tofacitinib. Three other patients achieved partial remission, one with elevated inflammatory markers and the other two with joint symptoms; these three patients also used fewer additional drugs, lower dosages of corticosteroids, and received less frequent injections of tocilizumab. Only one patient responded poorly to tofacitinib treatment, which manifested as a persistent elevation in CRP and ongoing symptoms of arthritis. These results suggest that the combination of tofacitinib and tocilizumab was safe and provided tangible clinical benefits for the management of pediatric Still's disease.

Huang *et al*. described an 11-year-old pediatric Still's disease patient who experienced adverse reactions to prolonged use of corticosteroids, including compression fractures, and was treated with 5 mg of tofacitinib twice daily for three months. Similar to our findings, this treatment led to complete remission of clinical symptoms and a lower disease activity score (20). Zhang et al. also described a patient with pediatric Still's disease who achieved complete clinical remission after sequential treatment with tocilizumab and tofacitinib. This patient initially received six bi-weekly injections of tocilizumab, and then five months of tofacitinib treatment, and then received a single dose of tocilizumab and 10 months of continuous tofacitinib therapy (21). Gillard et al. reported two patients with pediatric Still's disease who received JAK inhibitors (ruxolitinib and baricitinib) and achieved complete remission with reductions in corticosteroid dosages (22). He et al. reported the largest cohort to date (*n* = 7) of patients with refractory pediatric Still's disease who received JAK inhibitors. They reported that two patients achieved complete remission, two achieved with partial remission, and three had no response. He et al. also reported decreased use or complete discontinuation of corticosteroids in three patients, and complete remission of persistent arthritis in association with tocilizumab in two patients, partial remission in two patients, and no response in three patients (23). A possible reason for the higher remission rate in our study may be because none of our patients experienced MAS after initiation of tofacitinib, whereas the He et al. cohort included four patients with pediatric Still's disease-MAS (23). Combining our findings with previous studies, it is clear that tofacitinib has substantial therapeutic potential for treatment of pediatric Still's disease, because it can lead to clinical remission, a reduced dosage of corticosteroids, and a decreased frequency of tocilizumab injections. In our cohort, two patients presented with MAS at disease onset. Despite treatment with glucocorticoids, cyclosporine A, and tocilizumab, their CRP levels remained persistently elevated. Consequently, 1 month after starting tocilizumab, we introduced tofacitinib. Although, by current standards for treatment-sequence, this represents an earlier-than-usual use of tofacitinib, early introduction of this JAK inhibitor can be a viable option for refractory pediatric Still's disease. This is especially relevant in China, because anakinra was only approved on March 12, 2025 and its use for pediatric Still's disease is not yet covered by medical insurance, and canakinumab is not yet available. Furthermore, a study published this year in *Clinical Rheumatology* suggested that tofacitinib may be as effective as or even superior to biological and/or conventional DMARDs, because it leads to a faster and higher response rate in MAS patients without severe adverse events (24).

Our analysis of adverse reactions showed that only one patient experienced a minor upper respiratory tract infection, consistent with previous reports on the safety of tofacitinib (20–23). The incidence of adverse reactions was lower in our cohort than in the cohort of He et al. (23), likely because our patients received lower dosages of tofacitinib; only one of our patients (No. 2) received the full recommended dosage (>40 kg: 5 mg bid; 20–40 kg: 4 mg bid; 10–20 kg: 3.2 mg bid), and the others received significantly lower dosages (Table 2). Our patients received lower dosages because they were also using other potent medications, such as corticosteroids, cyclosporine, or tocilizumab, and we wanted to minimize the risk of severe infection. Nonetheless, even at these lower dosages of tofacitinib, our patients experienced significant benefits. Other reports suggested that higher dosages of JAK inhibitors might lead to higher a rate of clinical remission (22, 23). Therefore, further studies that compare the rates of clinical remission and adverse events following use of the full dosage of tofacitinib are warranted.

The main limitations of this study were that it only examined a small number of patients, it had a retrospective design, it did not include a control group, and it was performed at a single center. Our single-center retrospective design may also have been subjected to indication bias, because tofacitinib was reserved for patients with pediatric Still's disease cases who had refractory disease, and variations in follow-up duration led to some missing data. To address this, we applied last-observation-carried-forward for key outcome measures and performed a complete-case sensitivity analysis. Patients were censored at the last visit, and outcomes were reported at standard times (1, 3, 6, and 12 months) to ensure the robustness of our findings. Although our results provide preliminary support for the use of tofacitinib for pediatric Still's disease, more extensive clinical trials, particularly randomized controlled trials, are necessary to validate these findings and more comprehensively assess the benefits and safety of this drug. Thus, we eagerly anticipate the results of the ongoing Phase III clinical trials. Additionally, considering the importance of achieving normal growth and development in pediatric patients, future studies should also focus on the long-term outcomes.

## Conclusions

In conclusion, our study found that a combination treatment that includes tofacitinib can provide clinical remission in some patients with pediatric Still's disease, reduce their use of corticosteroids, and decrease the frequency of tocilizumab injections. We also found that low-dose oral tofacitinib was well-tolerated and associated with a low incidence of adverse reactions. However, given the limitations of this study, more extensive prospective studies are needed to validate these findings and assess the long-term benefits and safety of tofacitinib as a treatment for pediatric Still's disease.

## Data Availability

The raw data supporting the conclusions of this article will be made available by the authors, without undue reservation.
