# Supplementary material for: Tofacitinib as an adjuvant treatment for pediatric Still's disease
Source: Front Pediatr. 2025 Aug 20;13:1650675. doi: 10.3389/fped.2025.1650675 (PMC12405347; doi:10.3389/fped.2025.1650675)
Supplement: Supplementary file 1 [file Table1.docx]

**Supplementary Table 1.** Characteristics of patients at initiation of tofacitinib and the last follow-up.

|  | **Tofacitinib initiation** | **Last follow-up** |
| --- | --- | --- |
| Patient 1 | Fever  Leukocytosis: 10.82×10^9^/L  CRP: 77.3 mg/L  Ferritin: 959.7 ng/mL  ESR: 41 mm/h  AST and ALT: Normal | No symptoms  Leukocytosis: 6.06×10^9^/L  CRP: 1.9 mg/L  Ferritin: 27 ng/mL  ESR: 4 mm/h  AST and ALT: Normal |
| Patient 2 | Fever, Rash  Leukocytosis: 19.69×10^9^/L  CRP: 29.2 mg/L  Ferritin: 265.1 ng/mL  ESR: 100 mm/h  AST and ALT: Normal | No symptoms  Leukocytosis: 6.34×10^9^/L  CRP: 5 mg/L  Ferritin: 27.6 ng/mL  ESR: 4 mm/h  AST and ALT: Normal |
| Patient 3 | No symptoms  Leukocytosis: 12.98×10^9^/L  CRP: 171 mg/L  Ferritin: 237.2 ng/mL  ESR: 58 mm/h  AST and ALT: Normal | Arthritis  Leukocytosis: 11.51×10^9^/L  CRP: 118 mg/L  Ferritin: 137.2 ng/mL  ESR: 91 mm/h  AST and ALT: Normal |
| Patient 4 | Fever  Leukocytosis: 21.08×10^9^/L  CRP: 155 mg/L  Ferritin 213.3 ng/mL  ESR: 32 mm/h  AST and ALT: Normal | No symptoms  Leukocytosis: 9.01×10^9^/L  CRP: 5 mg/L  Ferritin: 93.2 ng/mL  ESR: 3 mm/h  AST and ALT: Normal |
| Patient 5 | Rash  Leukocytosis: 14.35×10^9^/L  CRP: 2.47 mg/L  Ferritin: 18 ng/mL  ESR: 2 mm/h  AST and ALT: Normal | No symptoms  Leukocytosis: 10.53×10^9^/L  CRP: 33.27 mg/L  Ferritin: 61.3 ng/mL  ESR: 18 mm/h  AST and ALT: Normal |
| Patient 6 | Arthralgia/arthritis, Rash, Lymphadenopathy  Leukocytosis: 12.1×10^9^/L  CRP: 76.5 mg/L  Ferritin: 143.6 ng/mL  ESR: 50 mm/h  AST and ALT: Normal | Morning stiffness  Leukocytosis: 4.34×10^9^/L  CRP: 5 mg/L  Ferritin: 35.7 ng/mL  ESR: 12 mm/h  AST and ALT: Normal |
| Patient 7 | Fever, Arthralgia/arthritis  Leukocytosis: 23.27×10^9^/L  CRP: 9.9 mg/L  Ferritin: 61.4 ng/mL  ESR: 4 mm/h  AST and ALT: Normal | Arthralgia  Leukocytosis: 6.36×10^9^/L  CRP: 1 mg/L  Ferritin: 22.5 ng/mL  ESR: 2 mm/h  AST and ALT: Normal |
| Patient 8 | Fever  Leukocytosis: 22.83×10^9^/L  CRP: 2.9 mg/L  Ferritin: 231 ng/mL  ESR: 9 mm/h  AST and ALT: Normal | No symptoms  Leukocytosis: 9.07×10^9^/L  CRP: 5 mg/L  Ferritin: 28.4 ng/mL  ESR: 2 mm/h  AST and ALT: Normal |
| Patient 9 | Slow growth  Leukocytosis: 10.05×10^9^/L  CRP: 1 mg/L  Ferritin: 23.6 ng/mL  ESR: 2 mm/h  AST and ALT: Normal | No symptoms  Leukocytosis: 8.3×10^9^/L  CRP: 5 mg/L  Ferritin: 8.3 ng/mL  ESR: 2 mm/h  AST and ALT: Normal |
| Patient 10 | Fever, Rash  Leukocytosis: 14.92×10^9^/L  CRP: 6.6 mg/L  Ferritin: 15000 ng/mL  ESR: 29 mm/h  AST and ALT: Normal | No symptoms  Leukocytosis: 6.15×10^9^/L  CRP: 5.14 mg/L  Ferritin: 27 ng/mL  ESR: 21 mm/h  AST and ALT: Normal |

CRP: C-reactive protein; ESR: erythrocyte sedimentation rate; AST: aspartate transaminase; ALT: alanine transaminase
